# Effect of a playful parenting programme on early childhood development and care outcomes of young children in vulnerable communities: findings from a quasi-experimental study

**DOI:** 10.1186/s12887-024-05161-8

**Published:** 2024-11-26

**Authors:** Viktoria Sargsyan, Harriet Walea, Bal Mahat, Robert Tamale, Ramdhani Chaudhary, Janet Birungi, Sabina Marasini, Nisha Thapa, Bihari Sharan Kuikel, Biraj Karmacharya, Muneera A. Rasheed

**Affiliations:** 1 World Vision Technical Support Office, Yerevan, Armenia; 2 World Vision Uganda, Kampala, Uganda; 3 World Vision Nepal, Lalitpur, Nepal; 4 Dhulikhel Hospital, Kathmandu University Hospital, Dhulikhel, Nepal; 5 University of Bergen, Bergen, Norway

**Keywords:** Early childhood development, Nurturing care, MDAT, Quasi-experimental

## Abstract

**Background:**

World Vision launched the Inclusive Playful Parenting for a Brighter Childhood (IPP4BC) project in identified vulnerable communities in Nepal and Uganda to mitigate risk for children at risk of poor development due to COVD 19. The intervention, based on the nurturing care framework, offered a customized parenting curriculum for young children, emphasizing holistic development through behavior change approaches tailored to local contexts.

**Objective:**

To evaluate the effect of the IPP4BC project, on early childhood development (ECD) and care outcomes of children under 6 years in vulnerable communities in Nepal and Uganda, particularly those affected by the COVID-19 pandemic.

**Methods:**

The intervention was delivered by trained facilitators over a 7-month period in different arms defined by the dosage: high (10 group sessions, 4 home visits) medium (5 group sessions, 2 home visits) or low (delivery of key messages through media) dose. An endline evaluation designed as quasi-experimental non-equivalent control groups post-test only study, assessed the effect of the project utilizing the Malawi Development Assessment Tool (MDAT) for child outcomes and the Multiple Indicator Cluster Survey (MICS) for caregiver outcomes. Additionally, an implementation survey was conducted to assess program fidelity and participant engagement.

**Findings:**

In Nepal, higher intervention doses were associated with significantly better child development outcomes (High dose M = 1.20, SD = 2.22, Medium dose M = 1.01, SD = 1.99; Low dose M = 0.43, SD = 2.32, *p* < 0.001) whereas in Uganda, only medium dose (M = -0.03, SD = 1.28) showed significant improvement (High dose M = -0.30, SD = 1.30; Low dose M = -0.28, SD = 1.51, *p* = 0.015). A higher proportion of caregivers in both high and medium dose reported engagement with early learning practices compared to low dose. With respect to uptake, in Nepal, a higher number of families received home visits compared to group sessions, while in Uganda, more families reported attending group sessions than receiving home visits.

**Conclusion:**

The study demonstrated that higher intervention doses were associated with better child development outcomes in Nepal and only with medium dose in Uganda, emphasizing the importance of implementation factors like dosage, quality and delivery modality in community-based interventions for improving ECD outcomes in vulnerable populations.

## Introduction

The COVID-19 pandemic posed significant threats to both health and family life, impacting approximately 1.38 billion children who lost access to schooling and childcare, which in turn hindered their educational and social activities and negatively affected their development [1, 2]. Additionally, the pandemic disrupted vital services for young children, including routine immunizations [3]. Parents faced heightened stress due to isolation, job losses, limited access to child support systems, and the overarching uncertainty of the situation, which significantly affected their mental health [4, 5]. These challenges were exacerbated in vulnerable communities where job losses were more severe, especially among daily wage workers, leading to increased stress for parents and a reduced capacity to provide nurturing care, thereby raising the risk of abuse [5–7].

These circumstances highlighted the urgent need for interventions to support parents in vulnerable populations during the pandemic [8]. To address these challenges, it was crucial to assist parents in adopting best practices for childcare. Some experts suggested that this period presented a unique opportunity for creative, playful parenting, which could counteract threats to children’s development [1]. The family environment is foundational for Early Childhood Development (ECD), where children’s early learning and growth take shape. Positive family interactions significantly influence children’s cognitive, emotional, and social development. Supportive family environments that are responsive and attentive contribute to better developmental outcomes [9]. The Lancet Series on ECD emphasizes the vital role families play in providing essential stimulation and care, asserting that “the family is the most important context for early childhood development” [10]. Engaging families in ECD initiatives not only enhances their capacity to support their children’s growth but also fosters resilience and strengthens community bonds. Several meta-analyses of interventions aimed to improve parental practices around stimulation and responsive interactions have shown benefits for children’s outcomes [11–14]. Furthermore, implementing contextualized caregiver programming is crucial, as it ensures that interventions are tailored to the specific cultural and social realities of families, thereby enhancing their effectiveness and relevance [15].

To address these challenges using a family-centered intervention, World Vision (WV) implemented the IPP4BC project, which aimed to integrate playful parenting into ongoing Education, Health, Nutrition, Multi-Faith Leader Engagement, Caregiver Skills Training, Advocacy, and Social Responsibility work in Uganda and Nepal. The project adopts a nurturing care model that encompasses nutrition, health, early stimulation, and responsive caregiving within an 18-month program. The study’s objectives focus on evaluating the effects of varying dosages of playful parenting interventions on child development and caregiving outcomes, specifically examining:

1. The impact of different dosages of integrated playful parenting interventions on:
  ◦ Child outcomes: motor skills, language, cognitive, and social-emotional development.
  ◦ Responsive parenting practices, early learning, and safety.
  ◦ Changes in caregiver mental wellbeing scores.
2. Evaluating intervention fidelity and quality.

## Methods

### Project site and participants

WV Nepal and WV Uganda identified geographic areas for implementation within 5 and 10 districts, respectively that had the greatest need and were significantly impacted by COVID-19. These areas had existing programs that were adapted during the pandemic to implement interventions related to education, health, nutrition, engaging multi-faith leaders, advocacy, and social accountability.

### Study design and sampling framework

**Fig. 1:**
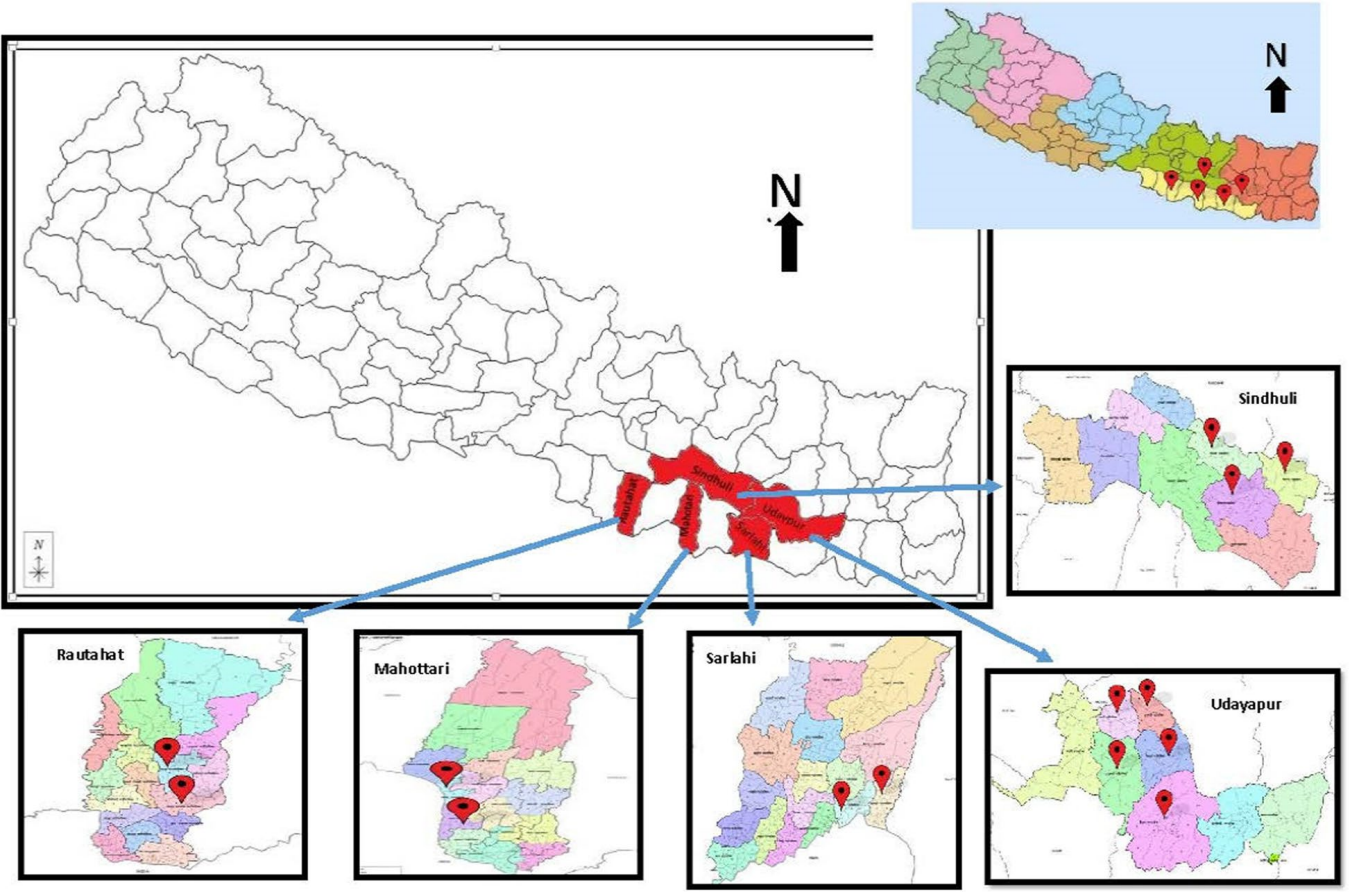
Study districts in Nepal

**Fig. 2:**
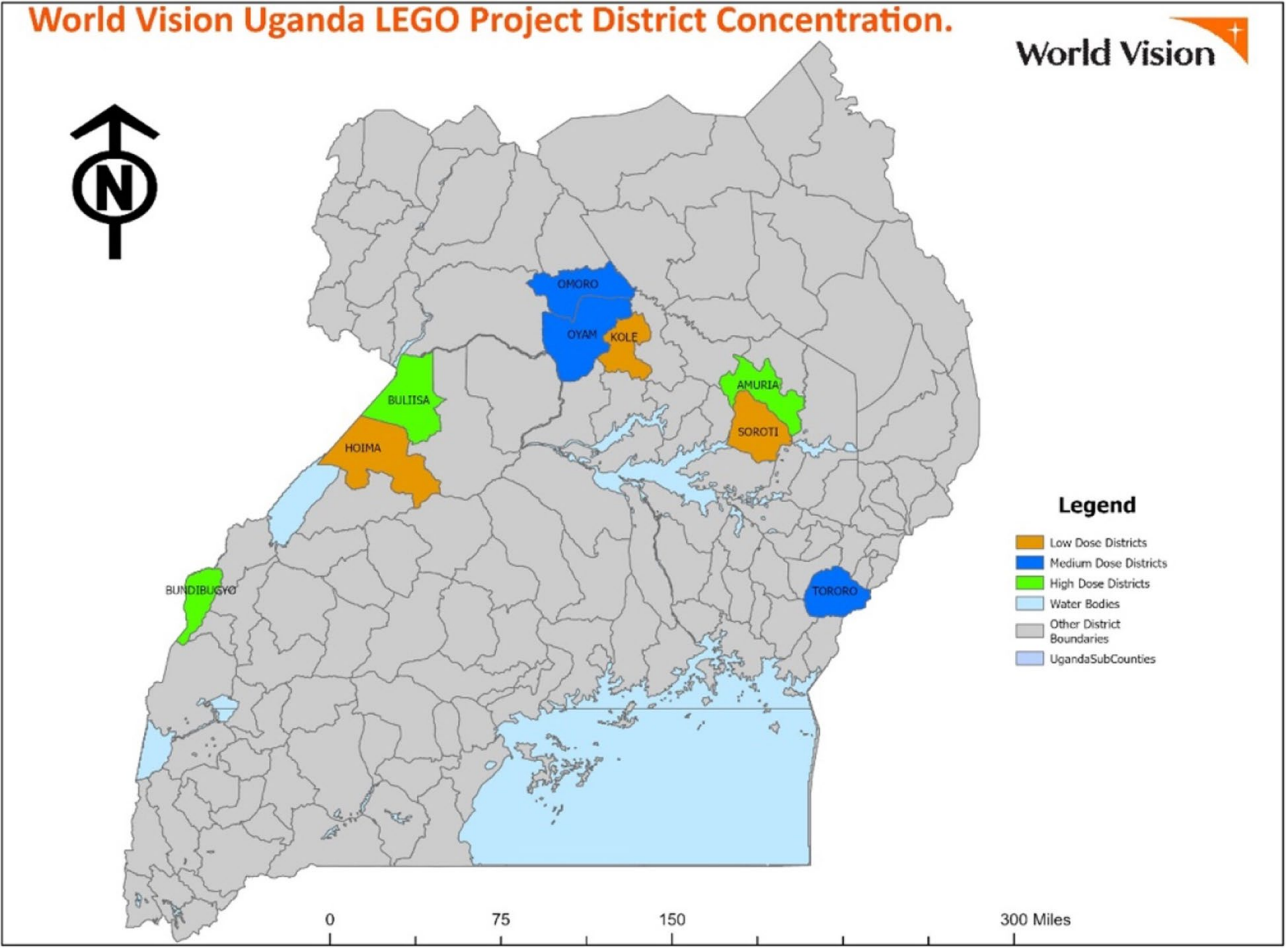
Study districts in Uganda

The evaluation was designed as a quasi-experimental nonequivalent control groups posttest-only design, employing a mixed-methods approach. The study comprised three arms: high dose, medium dose, and low dose; specific dosage details are provided in the intervention section. The quasi-experimental design was chosen due to logistical constraints in randomizing at the district or village level, ensuring operational feasibility while still allowing for comparisons across dosage groups. In Nepal, geographic areas within each district were allocated to 2 or 3 study arms (Fig. 1), whereas in Uganda, each of the ten districts were assigned to one study arm (Fig. 2). For outcome evaluation, the targeted sample was randomly selected from World Vision’s list of parents with children aged 0–60 months who were exposed to the IPP4BC interventions within the project target districts. The sample for each study arm (high, medium, and low) was further distributed proportionately according to the village population size to ensure statistical inference and representativeness in each village. Informed consent was obtained from each study participant prior to data collection.

### Ethics approval

Ethics approval was sought prior to data collection and was granted by the Nepal Health Research Council (No. 926) and the Uganda National Council of Science and Technology (No. SS1498ES).

### Sample size

The sample size was determined based on the primary outcome—early childhood development outcomes as indexed by scores on the Malawi Development Assessment Tool (MDAT) [16]. We employed the method of comparison of means between the intervention groups. The proposed sample size calculation was as follows:

$$n=\left\{2\ast\left(Z\alpha/2+Z\beta\right)^\wedge2\ast\sigma^\wedge2\right\}/d^\wedge2,$$

where ( Z_{α/2}) is the critical value of the Normal distribution at ( α/2) (for a confidence level of 95%, ( α) is 0.05 and the critical value is 1.96); ( Z_{β}) is the critical value of the Normal distribution at ( β) (for a power of 80%, ( β) is 0.2 and the critical value is 0.84); ( σ) is the population standard deviation, assumed to be a conservative value of 25 (from previous MDAT tool validations); and ( d) is the difference to be detected, assumed to be a conservative value of 5 score points. The calculation yielded a sample size of 422 for each arm of the study. We anticipate low attrition (estimated at 10%) and expect that most targeted families will remain in the area. The total sample size was thus 1,395, with 465 in each study arm. The sample was randomly selected from World Vision’s existing database of children aged 0–5/6 from the targeted communities, with each study arm’s sample further divided proportionately to the ward population size across the different municipalities, ensuring statistical inference and representativeness in each area.

### Intervention

The Go Baby Go (GBG) project model is a parenting curriculum designed to support caregivers of children aged 0–6 years by enriching their knowledge of child development and enhancing their parenting skills and self-confidence (https://www.wvi.org/publications/brochure/education/go-baby-go-caregiver-programme-early-childhood-development-0-6). The GBG curriculum is informed by the Nurturing Care Framework (NCF) and employs behavior change approaches such as experiential learning, empowerment, and appreciative inquiry, all while ensuring contextualization to meet the specific needs of communities. This model promotes key behaviors and practices essential for fostering healthy child development, emphasizing the importance of understanding how children’s brains develop across cognitive, emotional, and social domains. A central tenet of this model is responsive caregiving, which involves nurturing secure attachments, encouraging serve-and-return communication, and fostering positive, warm interactions between caregivers and children. By respecting and promoting a child’s agency, caregivers can support autonomy and build self-esteem. Additionally, the model advocates for early learning practices that stimulate curiosity and engagement through play and exploration. It places particular emphasis on caregiver mental well-being, encouraging self-care strategies to help manage stress and maintain a positive outlook. Recognizing the critical role of male caregivers, the Go Baby Go model includes two dedicated sessions aimed at increasing their understanding of ECD and enhancing their active participation in child play and communication, ultimately fostering a more supportive and interactive environment for children.

#### Skill-based group sessions

These sessions are delivered to primary caregivers of children aged 0–6 years through experiential activities conducted at the household cluster level, in accessible spaces identified by the communities. The sessions focus on holistic child development through the nurturing care framework, covering topics such as:

- Who we are as caregivers: the Go Baby Go journey
- Holistic child development
- Responsive caregiving
- Nurturing physical development
- Nurturing cognitive and language development
- Nurturing social and emotional development
- Play and toy creation
- Creating a conducive home environment
- Understanding that taking care of children is a family job
- Community action planning for embedding ECD programming within existing cultural practices

#### Home visits

Facilitators also conduct individualized home-based visits to caregivers, aimed at the following:

- Engaging with the most vulnerable families before the start of group sessions to discuss the program and identify any barriers to participation.
- Completing a minimum of 2 visits for vulnerability assessments with the caregiver, which informs the need and frequency of home visits, reinforces learning, facilitates in-depth dialogue through mentorship, develops a family well-being plan, identifies barriers to adopting best practices, and addresses additional family support needs, including referrals.
- Monitoring issues of care and protection during home visits, paying attention to caregiver mental health and well-being, and facilitating connections to key services.
- Conducting a final visit after caregivers finish group sessions to discuss ways to continue using GBG strategies at home.

#### Support networks

To facilitate sustainable support for families with young children, the model aims to:

- Build the workforce capacity of existing structures at the household cluster level and establish systems to replicate nurturing care practices beyond these clusters.
- Strengthen advocacy networks and improve services through Citizens Voice Action groups.
- Support families, particularly the most vulnerable, in connecting with key services available within and outside the intervention communities.

#### Intervention dosage

The intervention utilized three study arms, with different locations and districts receiving varying packages to address the research questions and understand how parenting practices influence child development by leveraging the nurturing care practices of caregivers.

### The high dose arms

This arm received the full GBG package:

**Table:** 

• 10 caregiver group sessions, with 2 additional sessions specifically for fathers (male caregivers) who had very limited time and opportunities to participate in the mothers’ sessions.
• A minimum of 4 complete home visits to provide individualized support for caregivers, with additional frequent visits to households with children at risk and those facing other vulnerabilities, including disabilities and mental health challenges among caregivers, among others.
• A minimum of 4 radio sessions led by community and district officials to disseminate messages on ECD, playful parenting, holistic child development, and nurturing care practices. In Nepal, 8 radio sessions were aired every Wednesday.
• Additional messaging through existing systems, such as Community Voice Action groups or village health teams in Uganda. In the Nepalese package, this was designated as messaging through Female Community Health Volunteers (health volunteer cadres under the Government of Nepal).

### The medium dose arms

This arm received a package modified GBG package.

**Table:** 

• 5 caregiver group sessions.
• A minimum of 2 home visits: the first visit primarily introduces the parenting program objectives and establishes relationships between caregivers and facilitators, while the final visit focuses on drawing actions and linking parents with existing programs.
• A minimum of 4 radio sessions led by community and district officials to disseminate messages on ECD, playful parenting, holistic child development, and nurturing care practices.
• Additional messaging through existing systems, such as Community Voice Action groups in Uganda.

### The low dose arm

This arm received a limited version of GBG.

**Table:** 

• There was no active engagement of caregivers by the project staff.
• Advocacy for improved parenting practices and high-quality ECD services was led by existing Citizens Voice Action groups within the communities, involving stakeholders from both local and higher government levels.
• A minimum of 4 radio sessions were conducted by community and district officials to disseminate messages on ECD, playful parenting, holistic child development, and nurturing care practices.

#### Delivery team

The GBG program was implemented by facilitators (GBGFs) specifically hired for the project. The community-led selection process involved local council leaders and included recommendations after consultation and verification. WV project staff finalized the list based on criteria such as relevant qualifications, adherence to child and adult safeguarding policies, and previous experience in community engagement. GBG Mentors were chosen from a pool of trained facilitators, selected for their enthusiasm, competence, and demonstrated facilitation skills during training. Selection criteria included post-test scores reflecting high performance and adaptability. Mentors provided ongoing support to facilitators, monitored sessions, facilitated reflections for improvement, conducted regular observation meetings, and reported to project staff. In Uganda, mentors co-delivered sessions as peer mentors, whereas in Nepal, their role focused more on supervision.

In Uganda’s high-dose group, there were 27 GBGFs and 5 mentors across 3 districts, while the medium-dose group included 25 GBGFs and 5 mentors in the same number of districts. In Nepal, the high-dose group had 33 GBGFs, and the medium-dose group had 40 GBGFs supported by 10 mentors. Co-facilitators were selected from among caregivers attending sessions, with mothers and fathers alternating in co-facilitating and sharing their experiences. Project officers (Pos) supported the mentors. In Nepal, the implementing partners were local NGOs, with each district having one partner NGO led by one PO. In Uganda, there were 4 Pos for the six intervention districts.

The strategy for the GBG parenting model aimed to maintain its core essence while integrating key messages into existing WV programs. This involved coordinating activities to ensure holistic care, particularly for most vulnerable (MV) families. The programs included health, early education, community groups, and multi-faith systems, allowing for a comprehensive approach that addressed various aspects of community life.

### Evaluation

The evaluation was conducted by external consultants at each site, with measures determined through discussions with the WV technical support team to ensure consistent data collection tools and methods for addressing shared research questions. Quantitative assessments measured child and caregiver/family outcomes as well as implementation data, while qualitative assessments involved caregivers, community facilitators, community leaders, and district stakeholders to evaluate the intervention’s impact and feasibility. These qualitative assessments focused on various aspects of implementation, such as recruitment (of caregivers and workforce), acceptability (relevance and satisfaction with interventions), reach, dose delivered and received, implementation fidelity, and quality (including workforce recruitment, training, retention, motivation, certification, minimum resources, and supervision). The end-line evaluation was completed between July and August in Nepal and Uganda, respectively, following 7 months of intervention delivery led by external research partners.

### Outcome and measures

#### Child development

We assessed child development using the MDAT [16], designed for children aged ≤ 5 years, focusing on four skill domains: gross motor, fine motor, language and listening, and social skills. The MDAT tool included 39 items for gross motor skills, 42 for fine motor skills, 40 for language and listening, and 36 for social skills. We calculated MDAT z-scores with the MDAT Scoring Application version 1.1, reporting the mean and standard deviation for both the overall model and individual MDAT domains.

#### Early learning and stimulation

To evaluate early learning and stimulation practices at home, we used six questions adapted from the Multiple Indicator Cluster Survey [17]. Caregivers were asked whether adults in the household had engaged in stimulating activities with children under six in the three days prior to the interview, with responses recorded as binary (yes/no) to indicate whether the child had participated in these activities.

#### Caregiver mental well-being

Caregivers’ mental well-being was assessed using the Warwick Edinburgh Mental Well-Being Scale (WEMWBS), a 14-item scale measuring feelings and functioning aspects of mental well-being [18]. Responses ranged from "none of the time" to "all of the time," with scores between 14 and 70. The 25th percentile was used as a cut-off to categorize caregivers’ mental well-being as poor (below 25th percentile) or good (above 25th percentile).

#### Implementation of the intervention

The endline evaluation survey included components on intervention participation and satisfaction. Additionally, process monitoring forms were developed to track implementation, including attendance records for each session. Quality checklists were created for GBG mentors to observe and rate sessions after group meetings and home visits.

#### Socio-economic and demographic characteristics

Socio-demographic variables included the child’s age, gender, religion, ethnicity, and parental education levels, along with family types. These questions were adapted from the baseline study tool.

#### Intervention experiences

Caregiver experiences were explored through qualitative interviews covering themes related to engagement in GBG sessions, home visits, and radio sessions, as well as satisfaction with the intervention components, the GBGF, and the overall program.

### Data collection and management

Prior collection, a team of enumerators was trained on the study objectives, ethics, and data collection tools (MDAT and the parents’ quantitative tool) by an external consultant. This training lasted five days at each country site. Quality control measures were implemented, including daily tablet checks by the survey data manager to ensure completeness and correct use of question skips by the enumerators. Data collection relied on World Vision area program staff to identify target communities (i.e., villages or wards) using World Vision’s household cluster approach, where 15–20 nearby households form a cluster for the re-enrollment of caregivers and children in the study. An ODK database was programmed into the tablets for data collection, which lasted approximately 15–20 days in both sites. Qualitative data was captured using recorders, and interviewers took notes to ensure no information was missed.

### Data analysis

Analysis was conducted using Stata version 16 by an external consultant. For normally distributed continuous variables, we utilized the mean and standard deviation for descriptive analysis, while median and interquartile range were employed for continuous variables with skewed distributions. Categorical variables were analyzed descriptively using frequency and percentage. Wealth quintiles were determined through principal component analysis (PCA). The primary outcome, child development, was presented as the mean of Developmental Adjustment Z-scores (DAZ). Extreme scores below -3 or above 3 were excluded. A Generalized Linear Model (GLM) was used to assess differences between intervention arms, adjusted for socio-economic status, family type, mother’s education, and child sex. A *p*-value of less than 0.05 was considered statistically significant. For process monitoring forms, project monitoring and evaluation officers analyzed data from each form and generated indicators based on the protocol for reach, dosage fidelity, and quality, under the guidance of the external consultant during a 5-day data analysis workshop.

For qualitative data, audio-recorded interviews were transcribed verbatim in English, a common language across all districts, with accuracy verified by cross-checking with the audio recordings. Transcripts were read multiple times before coding. Each transcript was coded by two individuals: the project coordinator and the consultant. Thematic analysis was conducted using a combination of inductive and deductive approaches to explore implementation. Initially, a codebook was developed based on deductive reasoning from interview guides and anticipated responses informed by the literature review, utilizing Proctor’s implementation outcomes to guide the coding process. Additional categories and codes were added inductively from the data, with some responses presented verbatim to support the findings. Narratives were then generated for each theme to address the study objectives.

## Findings

Table 1 presents the sociodemographic characteristics of the research participants, including the number of children under 5, number of adults in the household, primary caregiver, mother’s education, and SES tertiles across the three study arms for both sites. In Nepal, significant differences appeared in the number of children under 5 (higher in the low-dose group), SES (more poorest category in the medium-dose group), and maternal education (more mothers with no formal education in the medium-dose group). In Uganda, differences emerged in children under 5 and adults (higher in the high-dose group), primary caregivers (fewer mothers identified), and SES (more poorest category in the high-dose group).

**Table 1:** Sociodemographic characteristics of the research sample in Nepal and Uganda

	**Nepal**				**Uganda**			
	**Low Dose** *N*=246	**Medium Dose** *N*=609	**High dose** *N*=488	***p*-value**	**Low dose** *N*=451	**Medium dose** *N*=351	**High dose** *N*=380	*p*-value
Child age (Mean, SD)	34.1 (15.60)	35.90 (16.87)	36.8 (16.71)	0.11	39.50 (18.9)	38.83 (18.2)	37.52 (18.3)	0.30
Child age (N, %)								
< 3 years	156 (63.4)	338 (55.5)	269 (55.1)	0.068	212 (47)	169 (48.1)	195 (51.3)	0.45
4-6 years	90 (36.6)	271 (44.5)	219 (44.9)		239 (53)	182 (51.9)	185 (48.7)	
Child sex (N, %)								
Male	149 (60.6)	330 (54.2)	261 (53.5)	0.16	223 (49.4)	179 (51.0)	181 (47.6)	0.66
Female	97 (39.4)	279 (45.8)	227 (46.5)		228 (50.6)	172 (49.0)	199 (52.4)	
No. of children <5 in the household (Mean, SD)	2.73 (1.7)	2.69 (1.6)	2.48 (1.4)	0.039	1.74 (0.9)	1.6 (0.9)	1.9 (1.1)	0.0003
No. of adults in the household (Mean, SD)	6.73 (2.6)	7.03 (3.1)	6.81 (2.9)	0.28	5.9 (2.8)	6.5 (2.6)	7.2 (2.9)	<0.0001
Primary caregiver (N, %)								
Mother	241 (98.0)	580 (95.2)	467 (95.7)	0.18	374 (83.7)	300 (87.7)	314 (82.6)	0.008
Other	5 (2.0)	29 (4.8%)	21 (4.3)		77 (16.3)	51 (12.3)	66 (17.4)	
Mother’s education (N, %)								
No formal education	113 (46.1)	333 (55.1)	199 (40.9)	<0.001	14 (3.2)	22 (6.4)	29 (7.7)	0.015
Primary (1-8)	80 (32.7)	160 (26.5)	126 (25.9)		214 (48.0)	192 (56.0)	195 (51.5)	
Secondary or more	52 (21.2)	111 (18.4)	161 (33.1)		218 (48.8)	129 (37.6)	155 (40.8)	
SES tertiles (N, %)								
Tertile 1 (poorest)	66 (26.8)	229 (37.6)	154 (31.6)	0.001	142 (26.4)	148 (35.1)	174 (40.3)	<0.001
Tertile 2 (poor)	91 (37.0)	230 (37.8)	173 (35.5)		173 (32.2)	127 (30.1)	165 (38.2)	
Tertile 3 (least poor)	89 (36.2)	150 (24.6)	161 (33.0)		223 (41.5)	147 (34.8)	93 (21.5)	
Religion (N, %)								
Hindu/Christianity	215 (87.4)	487 (80.0)	366 (75.0)	<0.001	389 (86.2)	348 (99.1)	304 (80.0)	<0.001
Others	31 (12.6)	122 (20.0)	122 (25.0)		62 (13.8)	3 (0.9)	76 (20)	
Ecology								
Hilly	89 (36.2)	90 (14.8)	181 (37.1)		-	-	-	-
Plain	157 (63.8)	519 (85.2)	307 (62.9)	<0.001				

### Child development

In Nepal, children in the medium and high dose groups showed significantly higher mean scores in fine motor and cognitive domains (M = 0.69, SD = 1.29; M = 0.79, SD = 1.29), gross motor (M = 0.48, SD = 1.19; M = 0.67, SD = 1.19), language development (M = 0.34, SD = 1.44; M = 0.50, SD = 1.44) compared to the low dose group (M = 0.27, SD = 1.29; M = -0.13, SD = 1.18; M = 0.08, SD = 1.43) respectively. Additionally, significant higher score was found between high and medium dose for gross motor only (*p* = 0.017).

In Uganda, only children in the medium dose group showed significantly higher mean scores in all domains: fine motor and cognitive (M = 0.25, SD = 1.25), gross motor (M = 0.07, SD = 1.09), and language (-0.049, 1.21) compared to the low dose group (M = 0.09, SD = 1.17; M = -0.09, SD = 1.16; M = -0.52, SD = 1.29) respectively (Table 2). Similarly, children in the medium dose scored higher than high dose for all domains except social (Table 2).

**Table 2:** Mean and standard deviation of scores on domains of MDAT

	**Uganda**					
	**Low dose**	**Medium dose**	**High dose**	**Low Dose**	**Medium Dose**	**High dose**
	N, Mean (SD)	N, Mean (SD)	N, Mean (SD)	N, Mean (SD)	N, Mean (SD)	N, Mean (SD)
Total	201, 0.43 (2.32)	482, 1.01 (1.99)*	393, 1.20 (2.22)*	421, -0.28 (1.51)	329, -0.03 (1.28)*	354, -0.3 (1.3)^
Fine motor & cognitive	209, 0.27 (1.29)	511, 0.69 (1.29)*	393, 0.79 (1.29)*	398, 0.09 (1.25)	316, 0.25 (1.17)*	336, 0.06 (1.2)^
Gross motor	217, -0.13 (1.18)	545, 0.48 (1.19)*	441, 0.67 (1.19)*^	436, -0.09 (1.16)	334, 0.07 (1.09)*	371, -0.33 (1.12)^
Language	201, 0.08 (1.43)	482, 0.34 (1.44)*	358, 0.50 (1.44)*	439, -0.52 (1.29)	344, -0.49 (1.21)*	368, -0.51 (1.24)
Social	229, 0.07 (1.36)	554, 0.12 (1.37)	430, 0.13 (1.37)	443, 0.38 (1.31)	345, 0.02 (1.23)*	362, 0.41 (1.26)^

*MDAT* Malawi Developmental Assessment Tool

^*^p < 0.05 compared to low dose

^*p* < 0.05 compared to medium dose

### Early learning and stimulation

The data indicate that, in both Nepal and Uganda, the percentage of participants engaging in ≥ 4 stimulation activities increases with the dose of stimulation activities, moving from low to medium to high. In Nepal, engagement rises from 44.6% in the low-dose group to 49.7% in the medium-dose group, with a more substantial increase to 57.7% in the high-dose group. Similarly, in Uganda, the trend shows an increase from 64% in the low dose to 73% in the medium dose, followed by a further increase to 77% in the high dose (Table 3).

**Table 3:** Frequency and percentage of caregivers across different outcomes

	**Nepal**			**Uganda**		
	**Low dose**	**Medium dose**	**High dose**	**Low Dose**	**Medium Dose**	**High dose**
	*N* = 231	*N* = 569	*N* = 468	*N* = 451	*N* = 351	*N* = 380
Early leraning practices (4 or more in the past 3 days)	103 (44.6)	283 (49.7)	270 (57.7)*^	287 (63.6)	255 (72.6)*	293 (77.1)*
Responsive feeding practices (3 or more)	36 (14.6)	104 (17.1)	132 (27)*^	85 (18.9)	132 (37.6)*	36 (9.5)*^
Use of violent discipine (at least 1 in the past month month)	172 (69.9)	483 (79.3)*	314 (64.3)*^	363 (80.5)	268 (76.3)*	259 (68.2)*^
Maternal well-being (> 25th percentile)	184 (74.8)	443 (72.7)	403 (82.6)^	336 (74.5)	271 (77.2)	243 (63.9)^

^*^p < 0.05 compared to low dose

^*p* < 0.05 compared to medium dose

### Responsive feeding

In Nepal, 14.6% of caregivers in the low-dose group, 17.1% in the medium-dose, and 27% in the high-dose group practiced responsive feeding, showing a significant difference (*p* < 0.001) with the highest percentage in the high-dose group. In Uganda, the medium-dose group had the highest responsive feeding rate at 37.6%, followed by the low-dose at 18.9%, and high-dose at 9.5%, with a significant difference across all groups (*p* < 0.001) (Table 3).

### Child discipline

In Nepal, 69.9% of caregivers in the low-dose group, 79.3% in the medium-dose group, and 64.3% in the high-dose group reported using at least one violent discipline practice (*p* < 0.001). In Uganda, the data indicated that 80.5% of caregivers in the low-dose group, 76.3% in the medium-dose group, and 68.2% in the high-dose group reported using at least one violent discipline practice in the past month (*p* < 0.001) (Table 3).

### Caregiver mental well-being

In Nepal, the high dose had a positive effect, with well-being increasing from 75% (low dose) and 73% (medium dose) to 83% (high dose). In Uganda, well-being slightly increased from 75% (low dose) to 77% (medium dose) but dropped to 64% with the high dose (Table 3).

## Experiences

This section captures qualitative evidence from caregivers and the implementation team, highlighting themes of adoption, specifically: i) change in mothers, ii) change in fathers, and iii) change in the GBG workforce.

### Change in mothers

The GBG model has positively impacted mothers, including teenage mothers, through various transformative experiences.

- Value of learning through play: Mothers across all doses have embraced the significance of play. As one mother expressed, “*Ever since the program’s inception, I’ve seen that we have learned to play with our children for them to become bright*” [Mother, FGD, Tororo, Medium dose, Uganda]. This demonstrates the program’s role in enhancing maternal understanding of caregiving.
- Safe and stimulating environments: Mothers recognized the importance of using local materials to create safe, stimulating environments. One mother noted, "*Ever since the program started, I have organized cleaning days to remove hazards from my home*” [Mother, Tororo, Medium dose, Uganda].
- Building positive relationships: The program has fostered affectionate relationships between mothers and children, allowing for healthier interactions. One mother said, “*I have learned how to make play materials for my children, which has made them creative*” [Mother, Tororo, Medium dose, Uganda].
- Collective responsibility: The program emphasizes that child-rearing is a shared responsibility. As one mother in Uganda noted, “*Fathers have started playing with children these days*” [Mother, Medium dose, Uganda]. Another remarked, “*A child needs care from both father and mother for a better future*” [Mother, High dose, Nepal].

### Change in fathers

The GBG model has significantly influenced fathers’ perspectives and roles in parenting.

- Transforming child discipline: Fathers have moved away from corporal punishment, focusing instead on constructive discipline. A father shared, “*I no longer beat my children; I try very hard to ensure they get an education*” [Father, Amuria, High dose, Uganda].
- Active involvement in play: Fathers are more engaged in playful parenting, recognizing its importance for child development. One father stated, “*I participate in playing with my children because it’s crucial for their brain development*” [Father, Amuria, High dose, Uganda].
- Improved communication and relationships: Fathers are fostering deeper bonds with their children through more meaningful interactions. One mentioned, “*The GBG intervention has increased unity in the community*” [Father, Medium dose, Nepal].
- Monitoring child health: Fathers are more aware of their children’s health through play, understanding that changes in behavior can signal well-being or health issues. A father observed, “*When a child is not playing, it should signal that the baby is sick, and action has to be taken*” [Father, Omoro, Medium dose, Uganda].

### Change in GBG workforce

The GBG program has not only enriched the lives of caregivers but has also transformed facilitators and mentors.

- New knowledge: GBG facilitators have gained valuable insights into child development, advocating for the importance of free play. One facilitator noted, “*GBG taught me to allow children to play freely, instilling hope in parents*” [GBGF, Tororo, Medium dose, Uganda].
- Creating safe environments: GBG facilitators have learned to establish safe play environments by removing hazards and providing resources for children. “*I learned that there should not be stones where children play*” [TOT, Tororo, Medium dose, Uganda].
- Building relationships: The GBG program has empowered facilitators, enhancing their communication and counseling skills. One said, “*I can now speak in public without fear, thanks to the training*” [GBGF, Medium dose, Uganda].
- Promoting child rights: Facilitators have educated parents on children’s rights and the importance of early education, leading to positive changes in communities. “*Now parents send their children to school at a younger age*” [GBGF, Buliisa, High dose, Uganda].
- Collective inspiration: The GBG program has inspired not just individuals but entire communities, fostering a sense of unity. “*This intervention has strengthened my family relationship*” [TOT, Medium dose, Uganda].

In conclusion, the GBG program has led to significant improvements in parenting practices, family dynamics, and community involvement, resulting in more nurturing and attentive caregivers and healthier, happier children across diverse settings.

## Implementation evaluation findings

### Reach of the program

In Nepal, the high-dose intervention reached 1,900 participants in group sessions, whereas in Uganda, it engaged a significantly larger group of 33,061 individuals. Similarly, the medium-dose intervention in Nepal reached 2,415 participants, while in Uganda, it reached 17,072 individuals. Overall, Uganda had more participants in both the high and medium dose interventions compared to Nepal.

### Dose delivered: group sessions

**Fig. 3:**
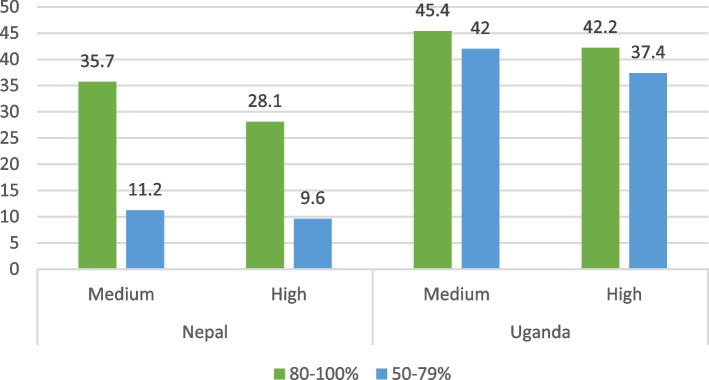
Percentage of eligible sessions attended by study site and intervention arms Note: Source of data for Nepal is group session attendance records and for Uganda it is endline survey

With respect to the dosage of group sessions, data from Nepal indicate that 36% of families attended 80–100% of group sessions (4–5 sessions) in the medium dose, while 28% of families in the high dose attended the same proportion of group sessions (8–10 sessions). In Uganda, for the medium dose intervention, 45.4% of caregivers attended 80–100% of the sessions, and 42% attended 50–79% of the sessions. Similarly, for the high-dose intervention, 42.2% of caregivers attended 80–100% of the sessions, while 37.4% attended 50–79% of the sessions (Fig. 3).

### Dose delivered: home visits

#### Percentage of targeted families receiving home visits

In Nepal, 95% of the targeted families in the medium-dose group received home visits, while this percentage increased to 98% in the high-dose group. In Uganda, only 50% of the targeted families in the medium-dose group received home visits, indicating notably low coverage. In the high-dose group in Uganda, the percentage of families receiving home visits was slightly lower at 72.3%.

#### Percentage of families receiving the full package of home visits

**Fig. 4:**
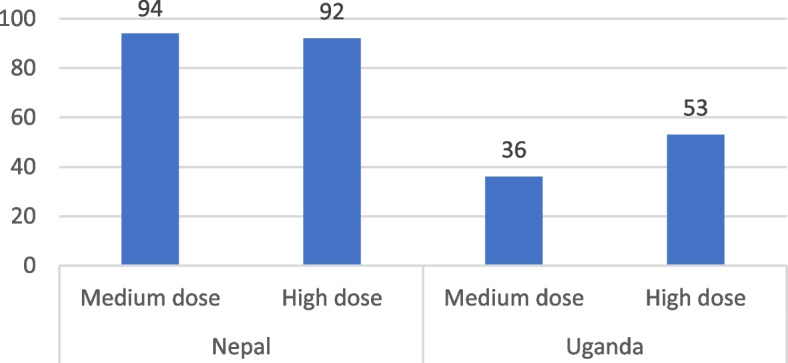
Proportion of caregivers receiving full package of home visits Note: Full package for High dose is 4 home visits and for medium dose is 2 home visits. Source of data for is endline survey

In Nepal, a high percentage of caregivers in both the medium (94%) and high (92%) dose groups received the full package of home visits, indicating strong coverage and adherence. In Uganda, there was a significant difference between the two dose groups, with only 35.6% of caregivers in the medium-dose group receiving the full package, which increased to 52.6% in the high-dose group. However, these proportions are considerably lower compared to those in Nepal (Fig. 4).

### Quality of the dose delivered

**Fig. 5:**
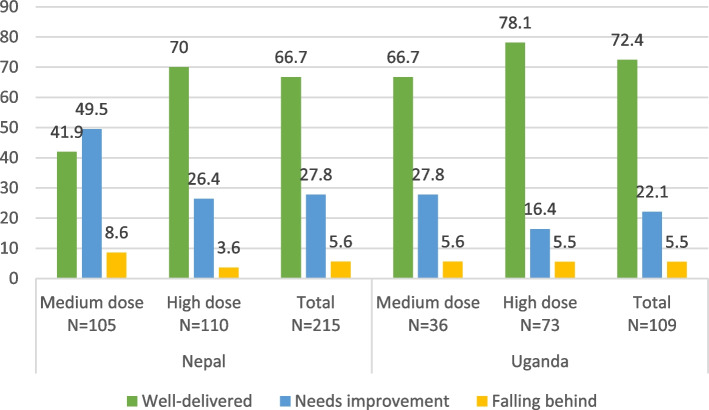
Quality of group sessions across intervention arms by study site

**Fig. 6:**
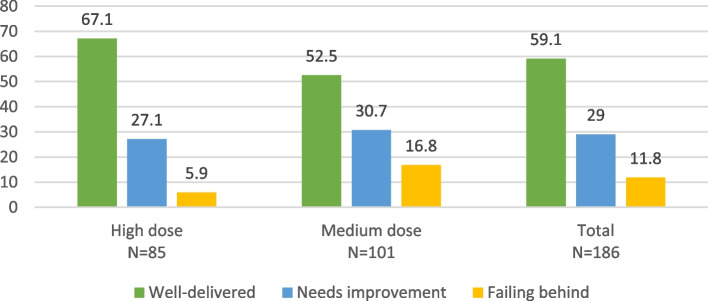
Quality of home visit across the intervention arms in Nepal

In Nepal, the high-dose program was well-delivered at a rate of 70%, with 26.4% needing improvement and 3.6% falling behind. For the medium-dose program, 41.9% was well-delivered, 49.5% needed improvement, and 8.6% was falling behind. In Uganda, the high-dose program achieved a well-delivery rate of 78.1%, with 27.8% needing improvement and 5.6% falling behind. For the medium-dose program, 66.7% was well-delivered, 27.8% needed improvement, and 5.6% was falling behind. On average, approximately 72.4% of the program was well-delivered, 27.8% needed improvement, and 5.6% was falling behind (Figs. 5 and 6).

Data on the quality of home visits was available only from Nepal. Analysis of the data from 186 households of caregivers indicated that more than half (59.1%) of the home visits were delivered effectively according to the guidelines, while 29.0% needed improvement, and 11.8% were identified as falling behind.

### Caregiver satisfaction

**Fig. 7:**
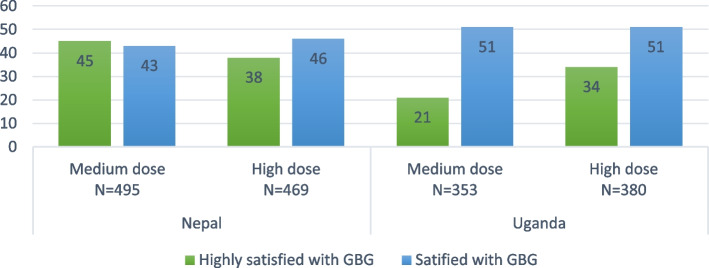
Proportion of caregivers highly satisfied and satisfied with GBG program Note: SOurce of data is endline survey

In both countries and across various intervention doses, a significant portion of caregivers expressed satisfaction with the GBG program, indicating that it has generally received a positive response from caregivers in both settings (Fig. 7).

### Feasibility of implementation

Experience with the implementation is categorized into three sub-themes: Facilitators, Challenges, and Recommendations.

#### Facilitators

Key enablers identified by participants include:

- Relevant content: Health workers noted the program’s alignment with their training, emphasizing the significance of play in childhood development. “*This project reinforced what we learned at school about play for proper growth*” [GBGM, Tororo, Medium dose, Uganda]. This awareness has led to increased community understanding of early childhood development.
- Community recognition: Encouraged by the program, community members are eager to engage, demonstrating its effectiveness. "*The majority of community members want to get involved, which motivates me to keep working*" [Project Officer, Paya and Mudumba, Uganda].
- Motivated by success stories: Witnessing transformations in families acts as a profound motivator. "*Parents have shared that their children are now healthy and strong, and their lives have improved*" [GBGM, Buliisa, High dose, Uganda].
- Professional development: Project officers have gained valuable skills that enhance their commitment to the program. “*The program has empowered me to better manage myself and my work*” [Project Officer, Amuria, High dose, Uganda].
- Valued by seniors: The program prioritizes feedback from stakeholders, reinforcing its collaborative nature. "*The program listens to our feedback for the betterment of the project*" [Faith Leader, Imam Asamuk, Uganda].

#### Challenges

Various stakeholders encountered distinct challenges during implementation:

- Non-responsive communities: Many fathers were reluctant to engage, leaving the burden of childcare on women. "*The fathers don’t participate, which creates challenges*" [Health Worker, High dose, Uganda].
- Cultural and economic barriers: Poverty significantly impacted parents’ ability to provide for their children. "*Poverty is the major impediment to proper child-rearing*" [Faith Leader, SDA, Uganda]. High poverty levels, compounded by climate change and limited awareness, hindered effective child development.
- Communication and weather issues: Technical problems and adverse weather conditions disrupted programs. "*Heavy rainfall affected participation, and mobile communication often fails during bad weather*" [GBGF, High dose, Uganda].
- Mentor challenges: The GBG team faced operational difficulties stemming from high costs, absenteeism, and inadequate communication among trainers. “*Absenteeism during learning sessions has affected our program coverage*” [GBGF, Tororo, Medium dose, Uganda]. Unpaid mentors expressed feelings of under-appreciation, which diminished their motivation [GBGM, Buliisa, Uganda].
- Lack of role clarity: Mentors reported confusion regarding their roles, leading to feelings of ambiguity: “*We didn’t know how the program was designed or where we fit in*” [GBGM, Buliisa, High dose, Uganda].
- Program management issues: Internal organizational challenges, budgeting constraints, and complicated reporting processes hampered implementation. "*Challenges ranged from a late start to high stakeholder expectations*" [Project Coordinator, IDI].

These challenges highlight the multifaceted obstacles faced in the GBG program, reflecting issues in healthcare, education, communication, transportation, and resource limitations. Addressing these challenges is essential for the program’s success and the well-being of the communities it serves.

#### Recommendations

The workforce identified key areas for improving the GBG parenting approach to ensure the program’s continuation, focusing on training, supervision, and continuous quality improvement (Table 4). Faith leaders emphasized the need for increased supervision and more comprehensive training to better support families. VHTs pointed out that extensive topics limit thorough training, recommending more focused sessions for in-depth understanding. GBG facilitators highlighted the importance of ongoing training, while CVAs suggested refresher sessions for teachers and caregivers to align with program goals. Additionally, GBG mentors proposed including local leaders and VHTs in reflection meetings to enhance community engagement. They called for intensified training efforts and an expansion of beneficiaries for greater impact. VHTs also recommended timely information sharing and participatory field report reviews for program improvements. Program officers suggested clarifying mentorship processes by identifying mentors at the sub-county or district levels.

**Table 4:** Quote for the recommendations theme

Theme	Quote
Recognize dedicated caregivers	*They should consider the caregivers because these caregivers started attending these sessions from the beginning up to the end, and they were disappointed when we were at the parenting days. They thought they would receive some gifts, but they were not given any. So, I would like them to consider how to motivate these caregivers.* [GBGM, KII, High dose, Uganda]
Program continuity	“*Continuity is essential to reach all community members, including those initially left out.*” [GBGF, FGDs, Buliisa, High dose] “*The project should be rolled out to all the villages in the Area Programme so that all children and mothers benefit from the interventions for positive parenting.*” [VHT, FGD, Amuria, High dose, Uganda] “*This project was done in 19 villages, so my recommendation is that they should scale up*.” [GBGM, Buliisa, High dose, KII, Uganda] “*The program should continue; removing hands off the program after one year is like leaving a one-year-old child to feed themselves*.” [PO, Izere Foundation, KII, Uganda] “*The engagement of fathers and other family members can be more effective as the decision-making power does not rely only on women*.” [GBGF, Medium dose, Uganda]
Investment in training supervision	*“We should invest more time in supervising program activities.”* [Faith leader, Omoro, Medium dose, KII, Uganda] *“More training is needed to improve our ability to support families*.” [Mentor, Tororo, Medium dose, KII, Uganda] *“The trainings were not enough to provide quality services because the topics were so wide, yet the training time was limited, affecting our ability to cover all the topics*.” [VHT, Amuria, High dose, FGD, Uganda] *“Continuous training is essential for maintaining our effectiveness.”* [GBGF, Buliisa, High dose, FGD] *“They should conduct refresher sessions to help the teachers get more knowledge and ensure they are applying what they were taught correctly.”* [CVA, Amuria, High dose, FGD] *“Reflectional meetings should be adjusted to include local leaders and VHTs, which could solve the challenge of mobilization and sensitization.”* [GBGM, Buliisa, High dose, KII, Uganda]
Data Management Improvement	*“They should improve on the system which they were using to receive our data*.” [GBGF, Buliisa, High dose, FGD, Uganda] *“Timely informing of field-implementation plans to ensure smooth program execution*.” [VHT, Bundibugyo, High dose, FGD, Uganda] *“Participatory review of field reports to identify areas for improvement and enhance program effectiveness*.” [VHT, Bundibugyo, High dose, FGD, Uganda] *“There was not clarity about the implementation at field level. We were also confused about the sessions and dosage. I feel there has been a communication gap between the plan and implementation*.” [Project officer, Rautahat, Nepal)
Commute assistance	*“VHTs be facilitated with transport means such as bicycles or motorcycles to ease their movements from home to home and this will increase number of home visits conducted*.” [VHT, FGD, High dose, Uganda]
Ownership from School Committee	*“We did receive the learning roots training and learnt about the playful engagement and learning to children but because of lack of teachers in the school, we need to teach other students as well.”* [ECD teacher, Rautahat, Nepal] *"Our local government doesn’t focus to ECD classes and teachers. They only look after higher classes. As this is a project with limited period, the local government should take ownership in provision of materials and trainings to us."* [ECD teacher, Mahottari, Nepal]
Stakeholder involvement	*“The program should bring on board more stakeholders and use different channels to communicate to its beneficiaries.”* [GBGM, Tororo, Medium dose, KII, Uganda] *“We would also wish to be involved throughout the project, right from the beginning other than only in the end.”* [ECD Focal person, Omoro, Medium dose, KII, Uganda] *“Let alone having engagements at the community level, I would say that the local government sector must be engaged since this is a sector which does supervision and monitoring of implementation, planning and decision-making, which includes budget allocation for local needs and priority areas.”* [Project coordinator, KII, Uganda] *“Overall, everything is great. But, if the local ward representative had prioritized this program for the development of the community, it would have been much better. Sometimes, I felt the ward representative is ignoring this program.”* [GBGF, Medium dose, Nepal]

*VHT* Village Health Team, *KII* Key Informant Interview, *ECD* Early Childhood Development, *CVA* Citizen Voice Action, *FGD* Focus Group Discussion, *GBGF* Go Baby Go Facilitator

## Discussion

The study aimed to evaluate the impact of a playful parenting intervention based on the GBG curriculum on the development and care outcomes of children under five, delivered in different dosages via group sessions and home visits. In Nepal, both high and medium dose groups showed significantly higher developmental outcomes than the low-dose group across all the MDAT domains, except for the social domain. In Uganda, the medium dose group achieved higher scores than the low-dose group across most domains (except for language), while the high-dose group surprisingly performed worse overall.

The lower MDAT scores, in Uganda’s high-dose group, despite greater engagement with play materials, could be influenced by baseline differences between the study sites. However, without baseline data, this remains speculative. Additionally, the high-dose group’s lower socio-economic status SES and poorer maternal well-being may have negatively impacted child development, as these factor can influence outcomes through various pathways. Another potential factor could be the difference in intervention quality; though the high dose group received a higher quality of group sessions, their overall outcomes were still poorer than the medium-dose group. These findings should be interpreted with caution due to study limitations. The small size limits generalizability, and the lack of standardized training for GBG mentors assessing facilitator performance may have introduced bias and variability in observations. Further research should allocate more time and resources to observer training and standardize data collection procedures to enhance reliability and accuracy.

Another noteworthy finding is that the overall scores in the Uganda were lower than those in Nepal, suggesting that children in Uganda may have been at a higher developmental risk. The absence of baseline scores limits our ability to fully interpret these differences across intervention groups. Interestingly, children had the highest scores on fine motor and cognitive domain across developmental domains in both study sites. This may be due to the MDAT assessment’s sensitivity to fine motor and cognitive changes or the intervention’s specific emphasis on the use of play materials, which likely enhanced these skills. A recent meta-analysis of 18 studies on the Reach-Up curriculum for young children found the greatest benefits for cognition, aligning with similar findings [19]. Improved cognitive scores via ECD interventions has also been reported in other meta-analysis of global interventions indictaing the emphaisis of the currcicula on cognition [11]. The current evaluation provides insights from programmatic implementation, demonstrating how lessons from real-world, non-controlled settings can guide effective scaling of interventions [20].

The results indicate that higher proportion of caregivers in the high and medium dose groups from both sides, reported engaging in play and stimulation activities over the past three days. This mirror findings from a humanitarian setting in Rwanda, where both medium and high dose using the GBG curriculum showed similar changes compared to the low dose group after 8 months of intervention [21]. However, there was no significant change observed in the use of violent disciplinary practices. A seven-month intervention period may be too short to induce noticeable changes in deeply ingrained practices.

The implementation evaluation reveal significant variations in the reach, dose delivered, and quality of the intervention across Uganda and Nepal. In Uganda, the group sessions had higher participation rates and quality, engaging more caregivers. On the other hand, in Nepal, home visits maintained greater fidelity, ensuring consistent delivery of content and dose. Similar attendance challenges have been noted in large-scale home visiting programs like Cuna Mas in Peru [22]. The program, Cuna Mas was started from scratch in 2012 and, within 3 years, was delivering weekly home visits to over 67,000 children in rural, disadvantaged areas. However, despite rapid expansion, Cuna Mas faced significant issues in maintaining consistent attendance.

This difference in delivery contributed to Uganda’s wider reach, driven by the group session format, while Nepal’s more intensive and individualized home visits led to lower reach. Despite this, child development outcomes were higher in Nepal, suggesting that personalized home visits, especially when GBGF workloads were lower, had a more substantial impact on child development This underscores a trade-off between reach and effectiveness: Uganda’s group sessions achieved greater scale, while Nepal’s home visits appeared to deliver better developmental outcomes. In a study from a rural community in India, where the Reach-Up curriculum, when delivered as group sessions was found to be more cost-effective and produced similar effects as home visits when compared within the same context [23]. However, the study was delivered as randomized controlled trial with similar sample sizes in the intervention groups while in Nepal and Uganda the sample sizes were quite different, and direct comparisons cannot be made.

Intervention designs need to recognize the importance of tailoring intervention methods to suit the needs of vulnerable populations. For such groups, home visits are a crucial consideration, as they offer a personalized approach that demands more resources but ensures equitable delivery. However, when the primary goal is widespread sensitization and demand generation, opting for group sessions may be more efficient. In cultures where group formats are less favored, integration into existing systems, such as mother and child health groups in Nepal, is a strategic approach. This integration requires more time and effort in engaging with stakeholders but fosters greater acceptability and participation, ultimately enhancing the program’s impact and effectiveness. Thus, the program’s design is flexible, adapting to the specific requirements and cultural nuances of each setting to achieve the best possible outcomes. In future, we envision GBG curriculum to be adapted for other vulnerable groups like children with neurodevelopmental disabilities given its emphasis on family [24]. We believe the most effective approach would be to integrate the core concepts of GBG with available programmes that are designed to support such children. The monitoring and evaluation will need to include tools that can screen for such impairments.

A key finding from the study is the strong commitment and engagement of project staff, leading to significant personal and professional growth. This demonstrates the program’s potential to empower its workforce while benefiting the target population. Many staff members have expressed a desire for additional training, supervision, and mentorship, highlighting the need for continuous development. Integrating these enhancements into the curriculum is vital for ensuring the program's sustainability and effectiveness. Keeping the above in mind, it is imperative to consider the integration of quantitative data collection methods. While the qualitative data has provided valuable insights into personal and professional changes, incorporating quantitative measures can offer a more comprehensive and standardized evaluation of the staff's impact on program outcomes and effectiveness. This data can aid in making evidence-based decisions, enhancing the workforce's performance, and ensuring the program's success in the future.

The study has several strengths. It focuses on implementation research, exploring how different doses optimize resource allocation in resource-constrained settings. Its holistic approach integrates the parenting model with health, education, and community systems, enhancing sustainability and real-world impact. The use of the evidence-based GBG model ensures interventions are grounded in established research, improving child development outcomes. Additionally, the study's large scale, spanning 15 districts across two continents, enhances the generalizability of findings. Conducting the study at two distinct sites allows for exploration of cultural and contextual variations. The use of multiple data sources, including surveys, interviews, and observations, provides a comprehensive view.

Limitations include delayed start, which shortened the intervention period and may have reduced its effectiveness. Design challenges, such as the lack of guidance documents and reliance on low-quality baseline data, weakened the evidence and hindered comparisons. Inconsistent process monitoring and the absence of standardized tools further affected the study's conclusions. Additionally, not assessing implementation in control arms missed insights on media usage, such as radio sessions. The study would also have benefitted from including other qualitative methods like direct observation to assess the impact of the interventions and how caregivers have bene able to implement the messages in their lives.

Based on the study findings, we present the following broad recommendations. Firstly, Invest in strategic program design by consulting experts and operations teams to ensure interventions align with best practices and are operationally feasible. Budgets should reflect equity by allocating resources to meet specific needs, especially in hard-to-reach communities, as seen in Uganda. Secondly, sustainability should be prioritized through strategies that emphasize continued engagement for long-term success. Leveraging social media platforms like TikTok can help create and sustain demand for interventions, while digital tools should be used for real-time supervision, monitoring, and maintaining fidelity to protocols. Finally, workforce engagement is critical, requiring adaptive management strategies informed by monitoring and evaluation (M&E) findings. Advancing implementation research is necessary to understand intervention effectiveness under varying conditions, with a focus on the quality of delivery and fidelity [25]. Strengthening M&E mechanisms through evaluator training and external assessments will improve program decision-making and outcomes.

We conclude that playful interventions can help mitigate the risk of poor development due to disruption in life such as the pandemic. However, implementation factors like delivery modality, dosage and quality are important factors to be considered when delivering interventions to improve child development outcomes and care outcomes.

## Data Availability

The datasets generated and/or analysed during the current study are not publicly available given the sensitive nature of information but are available from the corresponding author on reasonable request.
